# Self-Propagating High-Temperature Synthesis as an Enabling Route for High-Entropy MAX Phases

**DOI:** 10.3390/ma19091829

**Published:** 2026-04-29

**Authors:** Ali Haider Bhalli, Sofiya Aydinyan, Roman Ivanov, Irina Hussainova

**Affiliations:** 1 Department of Mechanical and Industrial Engineering, Tallinn University of Technology, 19086 Tallinn, Estonia; ali.bhalli@taltech.ee (A.H.B.); roman.ivanov@taltech.ee (R.I.); 2 Laboratory of Macro Kinetics of Solid-State Reactions, A.B. Nalbandyan Institute of Chemical Physics NAS RA, P. Sevak 5/2, Yerevan 0014, Armenia; sofiya.aydinyan@gmail.com

**Keywords:** SHS, combustion synthesis, high entropy, MAX phases, Mxenes

## Abstract

High-entropy MAX (HE-MAX) phases represent a new class of layered ceramics that combine the multi-principal-element chemistry of high-entropy materials with intrinsic damage tolerance, electrical conductivity, and multifunctionality of conventional MAX phases. Despite their promise, the synthesis of HE-MAX phases remains fundamentally constrained by sluggish multicomponent diffusion, narrow thermodynamic stability windows, and strong competition from thermodynamically favored binary and ternary carbides, borides, and nitrides. These challenges are further exacerbated by the volatility of A-site elements under near-equilibrium processing conditions. This review positions self-propagating high-temperature synthesis (SHS) as an energy-efficient, non-equilibrium processing route capable of stabilizing selected entropy-driven MAX chemistries through ultrafast thermal excursions and rapid quenching. A unified thermodynamic–kinetic framework is developed to elucidate the interplay among reaction enthalpy, configurational entropy, combustion wave sustainability, and phase evolution in HE-MAX systems. Predictions of thermochemical adiabatic temperature are systematically correlated with experimental SHS studies to delineate phase stability boundaries, stoichiometric sensitivity, and the roles of diluents and transient liquid formation. Finally, practical design principles for scalable SHS synthesis of HE-MAX phases are outlined, alongside strategies for their selective exfoliation into high-entropy MXenes and a critical assessment of their emerging functional applications.

## 1. Introduction

High-entropy ceramics have emerged as a transformative class of materials whose compositional complexity unlocks unprecedented combinations of structural robustness, chemical stability, and functional tunability. Among them, high-entropy MAX phases, which are essentially layered M_n+1_AXn compounds where multiple transition metals share the M-sublattice, represent a frontier with exceptional promise. More specifically, HE-MAX ceramics are layered ternary carbides and/or nitrides with a general formula (M_1_,M_2_,…,M_n_)_n+1_AX_n_, where multiple transition metals (M-site) are randomly distributed in near-equiatomic ratios, leading to high configurational entropy that stabilizes a single-phase solid solution. By merging the defect-tolerant, damage-resistant characteristics of MAX phases with the entropy-stabilized thermodynamics of multi-principal-element systems, these materials offer an unexplored design space for next-generation high-performance structural, electronic, and thermally resilient systems [1,2,3].

Recently, by leveraging entropy-driven phase stabilization, high-entropy MAX phases open pathways to exploring vast, previously inaccessible compositional spaces, offering tunable functionality for applications ranging from extreme environment structural components to energy storage, catalysis, and electronic devices [4,5]. Unlike conventional MAX phases, the incorporation of multiple transition metals in near-equiatomic ratios promotes single-phase formation with severe lattice distortion and heterogeneous bonding environments, leading to enhanced solid solution strengthening, modified phonon transport, and tunable thermal conductivity. Simultaneously, random M-site occupancy generates electronic structure complexity, including broadened density of states and localized bonding variations, enabling tailored electrical and catalytic behavior. The presence of diverse atomic environments also gives rise to sluggish diffusion kinetics, which enhances high-temperature stability, oxidation resistance, and phase retention. Moreover, synergistic effects may result in nonlinear improvements in mechanical and functional performance, while the altered defect chemistry contributes to improved damage tolerance and potential radiation resistance. These fundamental effects distinguish HE-MAX phases as a unique class of entropy-engineered, nano-laminated ceramics with emergent properties beyond those achievable in conventional systems [1,2,3,4,5,6,7,8]. These entropy-driven effects also offer a powerful route for designing precursors to multielement MXenes, enabling access to novel 2D chemistries with potentially enhanced stability and functionality [6,7].

However, despite their conceptual appeal, the practical realization of high-entropy MAX phases has been severely constrained. Conventional synthesis routes often struggle with the demanding kinetic landscape imposed by multielement diffusion, the propensity for competing phases, and the substantial thermal input required to drive long-range ordering [8,9]. Self-propagating high-temperature synthesis (SHS) offers a uniquely efficient and adaptable route for the synthesis of MAX phases and is particularly well suited to the rapid production of high-entropy MAX (HE-MAX) systems. This entropy-driven stabilization is especially relevant for systems with low adiabatic combustion temperatures, where conventional self-propagating high-temperature synthesis (SHS) may fail. In such cases, entropy contributions can compensate for insufficient reaction exothermicity, particularly when combined with thermally coupled reactions or aluminothermic reduction routes. The SHS method does not impose an intrinsic compositional limitation on HE-MAX phases; however, the accessible material candidate pool is defined at the reaction system level. In this context, the governing factor is the exothermicity of the overall reaction pathway, which depends on the initial mixture and reaction sequence rather than the nominal HE-MAX composition. For systems where the reaction is not sufficiently exothermic, thermochemical or kinetic activation strategies can be applied to enable combustion under a self-propagating mode.

**Figure 1:**
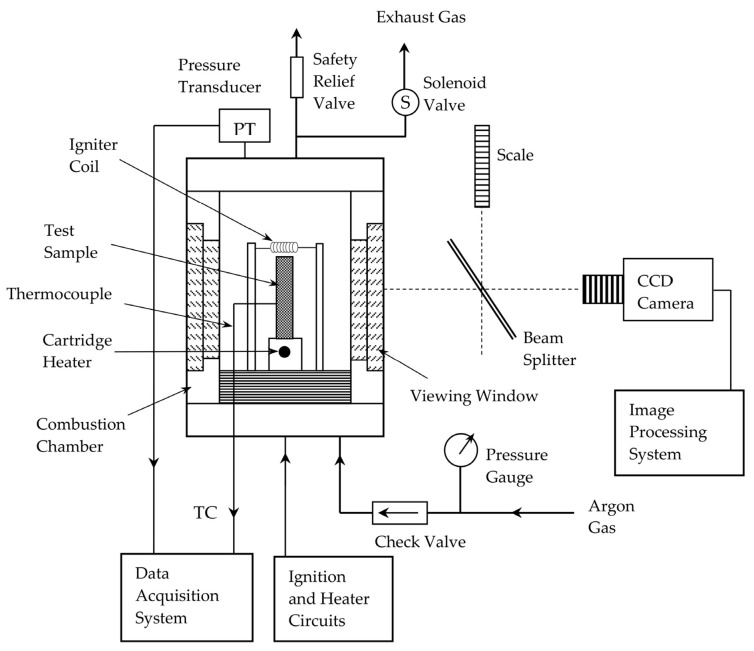
Schematic representation of the combustion-synthesis apparatus. Reproduced from Yeh and Chen [17] under CC BY 4.0.

The behavior of SHS reactions in these materials can be rationalized using a processing map that couples thermodynamic screening, such as composition and diluent content versus predicted adiabatic combustion temperature, with realistic, process-dependent heat losses arising from factors including green density, particle size, tooling contact, and ignition and boundary conditions. SHS operates as an autowave combustion process, in which locally ignited mixtures of fine powders generate a self-sustaining reaction front that propagates through the compact. Upon ignition at one end of a green body, an exothermic reaction is initiated and a thermal–chemical wave travels through the material, continuously transforming the reactants into the target phases, as schematically illustrated in Figure 1 [10]. SHS exploits highly exothermic reactions that, once ignited, propagate autonomously as combustion fronts capable of achieving ultrahigh temperatures, extreme heating rates, and nonequilibrium conditions unattainable by furnace-based approaches [11]. Since synthesis occurs in a few seconds, the intense thermal pulse promotes rapid interdiffusion, suppresses undesirable secondary phases, and potentially stabilize unconventional MAX phases far from equilibrium [12,13]. For example, the formation of carbides from refractory metals (e.g., Ta, Hf) is a highly exothermic, rapid reaction, leaving limited flexibility to regulate combustion parameters and to achieve the formation of HE MAX phase. Furthermore, SHS reactions are inherently scalable, enabling the production of bulk powders or coatings with unprecedented efficiency, and establishing a transformative, energy-efficient alternative to traditional high-temperature sintering processes [14,15].

Recent studies demonstrate that SHS can enable the rapid formation of high-purity high-entropy MAX (HE-MAX) phases. For example, a (Ti/Ta/V/Nb/Cr)_2_AlC (211) phase was synthesized via single-step combustion in an Ar atmosphere, exhibiting stable combustion wave propagation and yielding a well-defined layered MAX microstructure. Subsequent selective etching produced a single-phase high-entropy MXene with no detectable secondary phases or impurities [16]. Similarly, (Ti/Nb/V/Zr)_2_(S, SN)C (211) was synthesized by LMA-SHS in Ar using ~15 at% Sn served as a transient liquid binder. The reaction completed within seconds and resulted in ~98–99% phase purity, with only ~0.85 at.% Sn retained in the final product [15].

This review consolidates the current understanding of SHS as a transformative synthetic approach for high-entropy MAX (HE-MAX) phases and critically benchmarks it against conventional methods. The review focuses on how the SHS parameters, such as adiabatic temperature, reaction wave velocity, and compact density, affect phase formation, microstructural evolution, and compositional uniformity in multicomponent MAX systems. Analysis of reported HE-MAX systems reveals common challenges such as phase separation and volatility of the A-element and suggests potential solutions to counteract these unwanted effects. Furthermore, this review highlights the potential of SHS to accelerate the discovery of novel HE-MAX systems and enable the scalable synthesis of bulk powders and coatings with tailored properties. The obtained insights unlock the vast, largely unexplored HE-MAX compositional space and realize their potential for next-generation structural, electronic, and energy applications.

## 2. Fundamentals of SHS for MAX Phases

### 2.1. Principles of SHS

**Figure 2:**
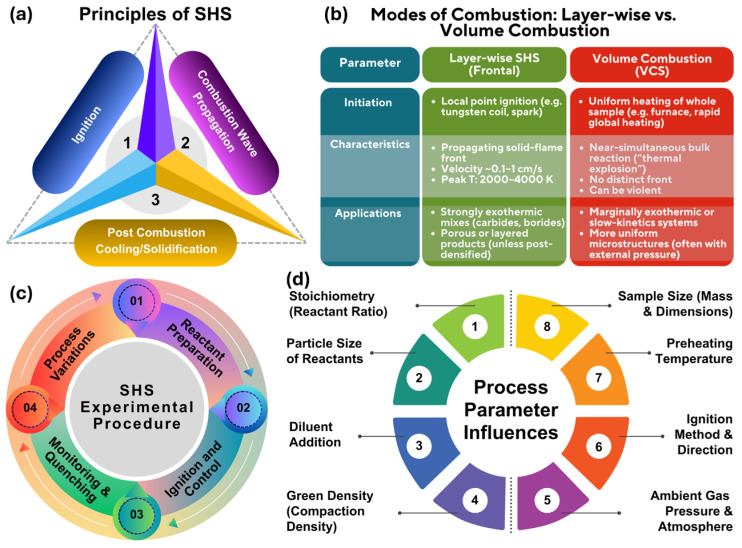
Summary of SHS process: (**a**) the core principles of ignition, wave propagation, and cooling; (**b**) modes of combustion; (**c**) the experimental procedure; and (**d**) critical parameters affecting the synthesis outcome.

Self-propagating high-temperature synthesis (SHS) is an autothermal, nonequilibrium processing route in which a locally ignited powder compact sustains a traveling combustion front (under certain conditions, undergoes volume combustion/thermal explosion). The core principles relevant to MAX and high-entropy MAX (HE-MAX) formation are as follows: (i) reliable ignition and attainment of a self-sustaining reaction; (ii) stability of front propagation and the associated temperature-velocity coupling; (iii) the ultrashort thermal cycle that governs intermediate reactions, phase selection, and microstructural refinement. Figure 2 summarizes these elements and highlights the key processing variables that control whether SHS yields high-purity MAX/HE-MAX products or mixed/porous reaction products.

**Figure 3:**
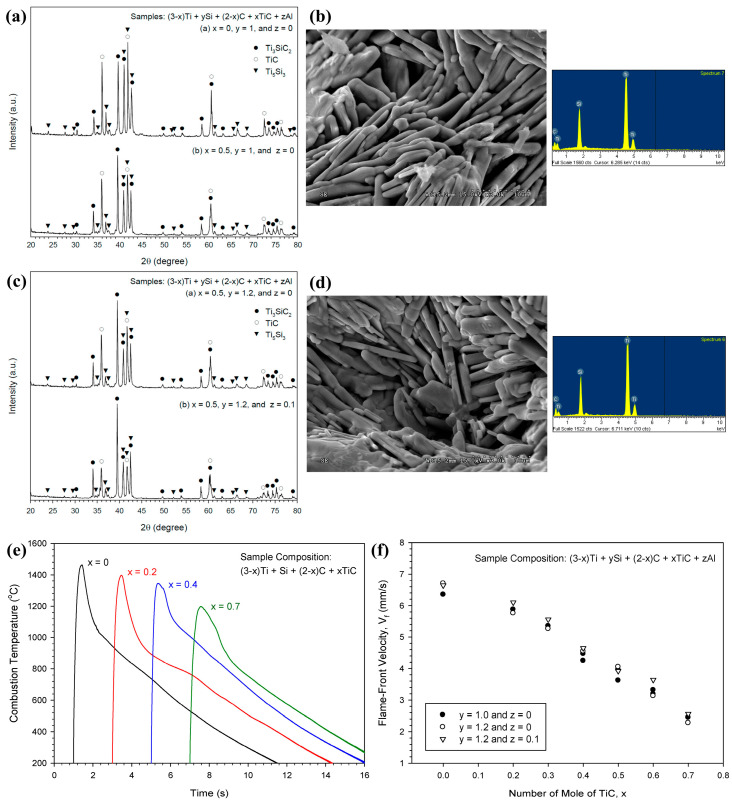
Phase evolution, microstructure, and combustion kinetics of Ti_3_SiC_2_ synthesized via SHS using TiC-containing reaction compacts: (**a**–**d**) XRD and SEM/EDS results for selected compositions derived from (3 − x) Ti + ySi + (2 − x) C + xTiC + zAl; (**a**,**b**) stoichiometric mixtures (y = 1.0, z = 0); (**c**,**d**) Si-rich (y = 1.2) and Si-rich/Al-added (y = 1.2, z = 0.1) mixtures; (**e**) combustion temperature profiles; (**f**) flame-front propagation velocity (V_f_) as a function of TiC (x) and stoichiometry adjustments (y = Si content; z = Al addition). Adapted from Figures 3–6, 9 and 10 of [19] under CC BY 4.0.

The thermal profile of SHS reaction is governed by the reaction exothermicity (reaction enthalpy) and by heat loss; self-sustaining combustion occurs when the heat-generation rate exceeds the heat-loss rate. Thermodynamic considerations and experiments show that combustion temperatures can rise sharply, typically reaching ~2000–3500 °C, depending on the reacting system [18]. MAX phases produced by SHS demonstrate a strong coupling between combustion temperature and flame-front velocity, both of which can be tuned through diluents and reactant stoichiometry. For example, in Ti_3_SiC_2_ synthesis, TiC additions reduce both the peak combustion temperature and propagation velocity, while a slight excess of Si and minor Al additions enhance phase conversion and yield, leading to the formation of the characteristic laminated MAX microstructure, as summarized in Figure 3 [19]. Similar trends are observed in Ti_3_AlC_2_ combustion synthesis, where TiC additions reduce the overall combustion exothermicity and decrease the reaction front, whereas compositional adjustments that promote liquid-phase-assisted transport lead to accelerated propagation [12].

During MAX-phase synthesis, transient liquid formation arising from low-melting metallic constituents may occur at an early stage and strongly influence the reaction pathways. The resulting molten phase can wet and redistribute along carbon or ceramic particles, thereby promoting rapid mass transport. Depending on the characteristics of the starting powders (e.g., particle size and porosity), phase formation may proceed predominantly via diffusion-controlled growth or through capillary-assisted infiltration mechanisms. In combustion-based routes, such as SHS, the reaction proceeds in the form of a localized combustion wave, leading to an abrupt temperature rise followed by rapid cooling. This highly localized heat generation enables short, high-temperature excursions that may favor MAX-phase formation while limiting prolonged diffusion and excessive grain coarsening [20,21]. SHS formation of MAX phases occurs via a sequence of fast, coupled intermediate reactions, and successful synthesis requires steering the reaction pathway to avoid competing intermediate phases.

The combustion wave velocity and front temperature are determined not only by the balance between heat release and heat losses, but also by reaction kinetics and the thermophysical properties of the reacting medium. In Ti_3_AlC_2_ SHS, a stoichiometric Ti:Al:C ratio of 3:1:2 sustains a flame-front velocity of ~5 mm⋅s^−1^ and Tc in the ~1280–1310 °C range, whereas excess Al (3:1.2:2) increases the velocity to 5.7–6.0 mm⋅s^−1^ due to the greater availability of molten Al that enhances reactant transport. In contrast, carbon-deficient mixtures exhibit reduced heat release and slow the propagation velocity to ~3.9 mm⋅s^−1^ [12]. In Ti_3_SiC_2_ SHS, TiC dilution markedly reduces both T_c_ and propagation velocity, demonstrating tunable SHS kinetics via diluents [19]. In aluminothermic SHS of MAX solid solutions, increasing the V_2_O_5_ fraction increases T_c_ and the combustion wave velocity for (Ti_1−x_V_x_)_2_AlC/Al_2_O_3_ (~1150 → 1550 °C; 1.4 → 4.8 mm⋅s^−1^) and (Cr_1−y_V_y_)_2_AlC/Al_2_O_3_ (~1200 → 1600 °C; 2.2 → 5.5 mm⋅s^−1^) due to the increasing contribution of the highly exothermic V_2_O_5_ + Al reaction [13].

MAX phases are typically synthesized under inert atmospheres (for carbides) or nitrogen-containing atmospheres (for nitrides and carbonitrides) to suppress oxidation and help maintain the intended composition; in systems where the A element is prone to loss, pressure-assisted SHS can improve A-element retention and preserve stoichiometry. Ignition is usually achieved by external heating (e.g., a heated coil or induction heating), and controlled ignition/heating conditions can improve combustion stability and microstructural uniformity [12,22].

A-site (e.g., Al) volatility constitutes a key destabilizing factor in Ti–Al–C MAX-phase SHS, as Al melts early in the reaction and exhibits a high vapor pressure, leading to evaporation-driven stoichiometric shifts that reduce Ti_3_AlC_2_ yield. This effect motivates the routine use of excess Al to offset volatilization losses. Consistently, high-temperature exposure of Ti_3_AlC_2_ under inert or vacuum conditions promotes surface decomposition toward TiC_x_ with concomitant evolution of gaseous Al, indicating volatility-driven instability at elevated temperatures [12,23]. In addition to composition, the processing atmosphere and pressure influence densification and microstructural evolution, particularly in systems involving volatile species or gas-phase transport, whereas phase purity is primarily governed by the starting stoichiometry (including deliberate A-site excess) and additive selection. These considerations underpin practical SHS strategies such as 5–20% A-site excess, encapsulation foils, and rapid post-front quenching to preserve the target composition and phase assemblage [13,24].

For MAX-phase synthesis, argon backfills are widely used to suppress oxidation and to buffer heat flow, while vacuum or sealed tubes help to retain volatile A-elements such as aluminum and silicon [25]. In combustion synthesis of MAX phases, applying a short-duration moderate uniaxial pressure during thermal explosion (“reactive forging”) can reduce porosity and improve consolidation, provided pressure is applied while a liquid/soft fraction is present. Thermal explosion without pressure yields porous Ti_2_AlC, whereas reactive forging at ~30 MPa can produce >98% dense material, and Ti_3_SiC_2_ based products can reach ~95% relative density under higher loads. Because applied pressure and tooling contact enhance heat extraction and can affect combustion stability, pressure-assisted TE–SHS routes are typically conducted at pressures on the order of tens of MPa, with careful control of pressurization timing to avoid premature quenching of the reaction. Together, propagating SHS and pressure-assisted thermal explosion modes provide complementary flexibility in designing SHS routes for MAX phases, particularly for balancing A-site volatility control with targeted microstructural development and densification [13,26].

Microstructural parameters of the starting mixture play a central role in governing SHS behavior. Particle size strongly influences reaction kinetics by altering the available interfacial area and diffusion distances. Finer particles provide a larger specific surface area, increase the number of reactive contacts, and facilitate interdiffusion between constituents. As a result, ignition temperature may decrease and combustion velocity may increase. In contrast, coarse particles can limit contact area and slow down interfacial reactions, potentially leading to incomplete conversion or heterogeneous phase formation.

This general trend is supported by reported MAX-phase SHS studies. For example, for Ti_2_AlC, combustion synthesis that changing Ti and Al particle size had only a limited effect on the phases formed, but it measurably altered the green density of the compact and the porosity of the final product; in contrast, the carbon source had a much stronger influence on phase evolution. Using finer, better-dispersed graphite or lamp black favored Ti_2_AlC + TiC formation, whereas larger carbon fibers, which reduced carbon dispersion and increased direct Ti-Al particle contact, promoted incomplete reaction and the formation of Ti_3_Al + TiC instead of Ti_2_AlC. Their graphite-based compacts also exhibited higher green density than carbon-fiber-based compacts, and the resulting products were less porous [27]. A similar raw material effect was reported in [28] for MA-SHS of Ti-Al-C MAX phases: TiC (<100 μm) as the carbon source favored high-purity Ti_3_AlC_2_ (up to 99.26 wt.%), whereas activated carbon (<10 μm) promoted Ti_3_AlC_2_-Ti_2_AlC dual-MAX formation and produced smaller crystal sizes than the TiC-derived route. It was demonstrated that, in the Ti–Al–C system, mechanical activation (MA) leads to an increased formation of TiC, whereas the substitution of Ti with TiH_2_ facilitates the formation of the Ti_3_AlC_2_ MAX phase [29,30]. Moreover, when comparing the plate-like grains of Ti_3_AlC_2_ obtained from elements and the Al_4_C_3_-containing sample, one may note that, due to the lower combustion temperature, Al_4_C_3_ contributed to the formation of slightly finer laminates with a size of 5–10 µm [31]. These examples illustrate that raw material characteristics do not merely affect ignition behavior, but can directly shift phase composition, compact packing, porosity, and final microstructure.

Green density represents another critical parameter. The degree of compaction controls porosity, pore connectivity, and therefore the effective thermal conductivity and heat capacity of the compact. Higher green densities typically enhance conductive heat transfer, enabling more effective preheating of the reactants ahead of the combustion front and promoting stable wave propagation. However, excessively high density may reduce gas permeability in systems where gaseous species participate in mass transport. Conversely, high porosity decreases effective thermal conductivity and may result in unstable or quenched combustion.

Macrokinetic analyses of Ti-based systems, including Ti + C mixtures commonly used as model systems, have demonstrated a systematic dependence of combustion velocity on titanium particle size. These observations are generally interpreted within convective–conductive combustion frameworks, which couple heat transfer, reaction kinetics, phase evolution, and, where relevant, gas transport within the porous medium. Such models provide a useful basis for understanding and predicting SHS behavior in more complex multicomponent systems, including those used for MAX-phase synthesis [13,32]. Finally, stoichiometric balance is critically excess or deficiency in reactants alters the exothermicity, which in turn affects the combustion temperature and wave velocity. In practical MAX-phase synthesis, a slight excess of the A-element is commonly used to compensate evaporation, but large deviations from stoichiometry hinder self-sustaining combustion [12,19]. Table 1 summarizes commonly reported qualitative trends in SHS/TE synthesis of MAX and HE-MAX phases, showing how insufficient or excessive reaction intensity and heat loss shift the outcome from quenched fronts and mixed phases to stable propagation and higher phase purity.

Table 2 lists key SHS control parameters and typical consequences when conditions move outside the stable propagation window; the specific boundaries depend on composition, compact geometry, and thermal boundary conditions.

### 2.2. Thermodynamic Considerations

In SHS of MAX and HE-MAX phases, the principal thermodynamic driver is the reaction enthalpy, as it directly governs the adiabatic combustion temperature (T_ad_). Under adiabatic assumptions, T_ad_ is determined by the balance between reaction exothermicity and the sensible/latent heat of the products and any diluent phases. Therefore, highly exothermic reactions yield elevated T_ad_, whereas the presence of diluents and unavoidable heat losses act to suppress the combustion temperature [12,19]. For MAX-phase systems, however, an excessively high T_ad_ can be detrimental because it may shift the equilibrium toward thermodynamically stable binary carbides and lead to decomposition of the target MAX phase; therefore, thermal management is important to keep the reaction temperature within the MAX-phase stability window [36].

A well-established empirical criterion in combustion synthesis is that gasless SHS requires T_ad_ ≥ 1800 K for the reaction front to propagate without autonomously. If T_ad_ falls below this threshold, the combustion front may become unstable or fail to remain self-sustaining, unless the reactant compact is preheated or supplementary heat is supplied [37]. Although SHS is often approximated as a quasi-adiabatic process, in actual peak temperature (T_p_ or T_c_) in real systems is typically lower than the adiabatic temperature T_ad_ due to heat loss to the surroundings highlights the necessity of sufficient reaction enthalpy to offset such thermal dissipation and ensure a self-sustaining combustion front. High-entropy MAX compositions gain an additional thermodynamic stabilization from configurational mixing entropy. At the elevated temperatures present at the SHS reaction front, the −TΔS_mix_ contribution becomes sufficiently large to lower the Gibbs free energy of the multicomponent solid solution, thereby expanding the single-phase stability region and suppressing segregation into competing high-entropy carbides or alloys. This entropy-driven stabilization is well documented in high-entropy ceramics and provides the primary thermodynamic rationale for employing SHS to synthesize complex HE-MAX phases within ultrashort thermal spikes [37,38].

**Figure 4:**
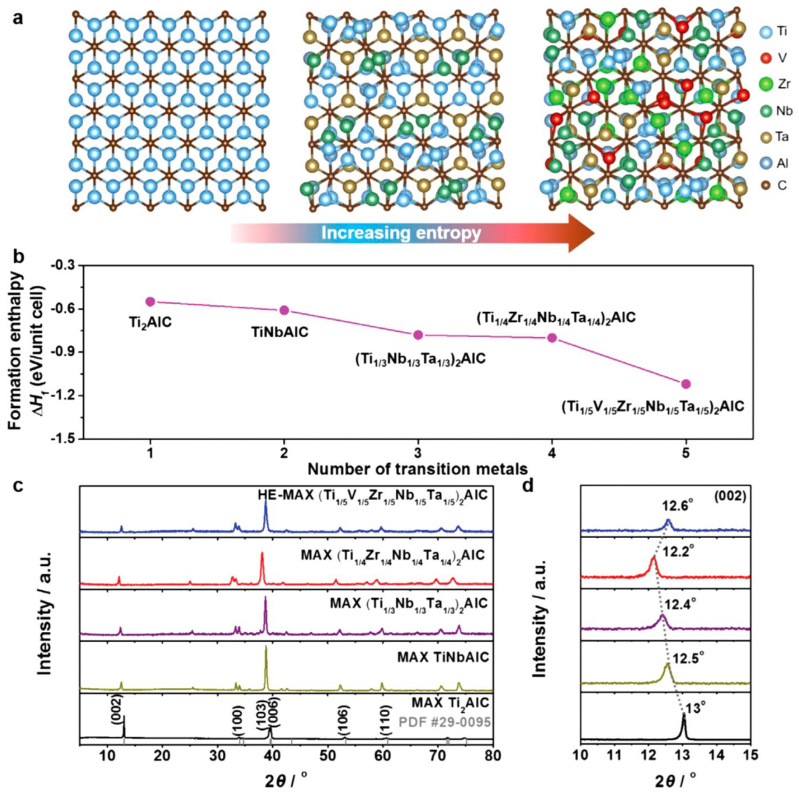
Theoretical and experimental evidence for the formation and stability of HE-MAX phases: (**a**) schematic diagram illustrating severe lattice distortions in the crystal structure as configurational entropy increases with multielement substitution at the M-site; (**b**) density functional theory (DFT) calculations for the (Ti, V, Zr, Nb, Ta)-system; (**c**,**d**) corresponding experimental X-ray diffraction (XRD) patterns confirming the formation of MAX phase structure. Reproduced with permission from [38]. Copyright 2021 Wiley-VCH.

First-principles predictions together with XRD evidence further support entropy-assisted MAX stabilization, showing reduced formation enthalpy and systematic lattice expansion as compositional complexity increases (Figure 4) [38].

Despite favorable thermodynamics, the success of SHS is critically governed by kinetics. Sustained reaction propagation requires efficient transport of both heat and matter along the combustion front. Insufficient heat release or excessive heat-sink effects can destabilize the front, leading to non-steady, pulsating, or fully extinguished combustion. For instance, in Ti-Si-C systems, the addition of inert TiC reduces both the adiabatic combustion temperature and the front velocity, thereby suppressing MAX-phase formation. In contrast, a small stoichiometric excess of Al produces a transient liquid phase that enhances mass transport and promotes more complete conversion. SHS feasibility therefore emerges from the coupled interplay of enthalpy (heat generation), entropy (phase stability), and kinetics (the ability to sustain a propagating reaction front) [39,40].

In practice, reaction intensity in SHS is commonly tuned through stoichiometric adjustments. For instance, a ~10–20% Al excess in Ti-Al-C introduces a transient liquid Al phase that increases combustion temperature/velocity and improves Ti_3_AlC_2_ yield [41]. Conversely, the addition of “thermal ballast” diluents can moderate overly vigorous reactions by acting as a heat sink and slowing the combustion wave. In Ti-Si-C, increasing TiC content has been reported to reduce the peak temperature (e.g., from ~1464 °C to ~1200 °C) and decrease wave velocity; an intermediate TiC fraction yields the highest Ti_3_SiC_2_ content, beyond which further TiC addition suppresses phase formation as the temperature falls below the optimal window [19]. Endothermic additives can exert a similar moderating effect; for example, TiH_2_ decomposition absorbs heat and can markedly reduce combustion temperature and propagation speed [12].

**Figure 5:**
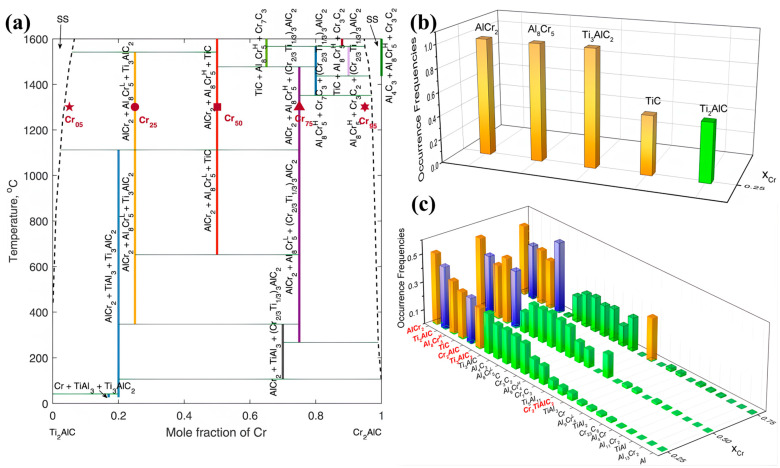
CALPHAD and first-principles prediction of phase stability in the Ti_2_AlC-Cr_2_AlC pseudo-binary system: (**a**) deterministic pseudo-binary phase diagram; (**b**) phase-occurrence frequencies obtained from stochastic Gibbs energy minimization at 1300 °C under ± 5% Al/C stoichiometry uncertainty; (**c**) full stochastic phase-occurrence probability distribution across compositions. Adapted from Figures 2–4 of [42] under CC BY.

In multicomponent HE-MAX systems, exploring the full composition space experimentally is challenging, so CALPHAD-based thermodynamic calculations are commonly used to screen viable SHS mixtures and flag likely competing phases. In the context of SHS, however, only reactant mixtures with sufficiently high exothermicity can sustain a self-propagating combustion front, making reaction enthalpy a primary constraint on feasible compositions. Within this subset of thermodynamically viable mixtures, CALPHAD provides a critical framework for mapping the stability domains of HE-MAX phases and identifying potential competing phases, thereby enabling rational design and predictive synthesis of complex high-entropy MAX systems. First-principles–informed Bayesian CALPHAD calculations show that tiny deviations from stoichiometry or slight loss of A site may drive the product toward a multiphase mixture in SHS, consistent with experimental observations. These findings highlight that careful composition selection and stringent control of A-site element volatility are essential for the reliable synthesis of HE-MAX phases. The sensitivity of SHS feasibility to reaction enthalpy and stoichiometry is quantitatively illustrated for the Ti_2_AlC-Cr_2_AlC pseudo-binary system through thermodynamic and first-principles predictions (Figure 5), which reveal narrow solid solution stability windows surrounded by extensive competing carbide and intermetallic phase fields. Stochastic Gibbs energy minimization further shows that small Al/C deviations at SHS temperatures can shift the dominant phase assemblage, rationalizing the experimentally observed variability in MAX and HE-MAX formation [42].

For SHS, CALPHAD datasets can be coupled with the SHS energy balance to calculate T_ad_ as a function of stoichiometry and diluent fraction [43]. When T_ad_ is close to the ~1800 K threshold, the calculation can indicate whether adjustments are needed, such as increasing the A-site element to create transient liquid, reducing diluent content, or preheating the compact [44]. An additional practical route to limit volatility and oxidation during SHS is molten-salt shielding/sealing (e.g., NaCl,KCl), where the salt acts as a physical barrier and high-temperature medium that can reduce material loss at elevated temperature and enable high-purity MAX powders even when processing in air [45].

### 2.3. Kinetic and Microstructural Design Principles

Whereas thermodynamics determines the possibility of a reaction, kinetics governs its actual realization by controlling its rate, conversion, and microstructure. In SHS, the combustion front propagates rapidly; therefore, successful HE-MAX formation depends on balancing heat generation with mass transport and reaction sequencing within a very short high-temperature time window.

Consequently, the initial powder and compact condition is a key kinetic lever. SHS studies on high-entropy systems show that phase assemblage is substantially more sensitive to stoichiometry than in conventional MAX phases. For instance, during combustion synthesis of the (Ti, Ta, V, Nb, Cr)_2_AlC solid solution system, stoichiometry deviations do decrease overall yield and change the phase pathway; stoichiometric mixtures prefer high-entropy carbides, and the fine-tuning of Al and C is necessary to stabilize the layered HE-MAX phases and perfectly avoid mixed 211/413 intergrowths [16].

**Figure 6:**
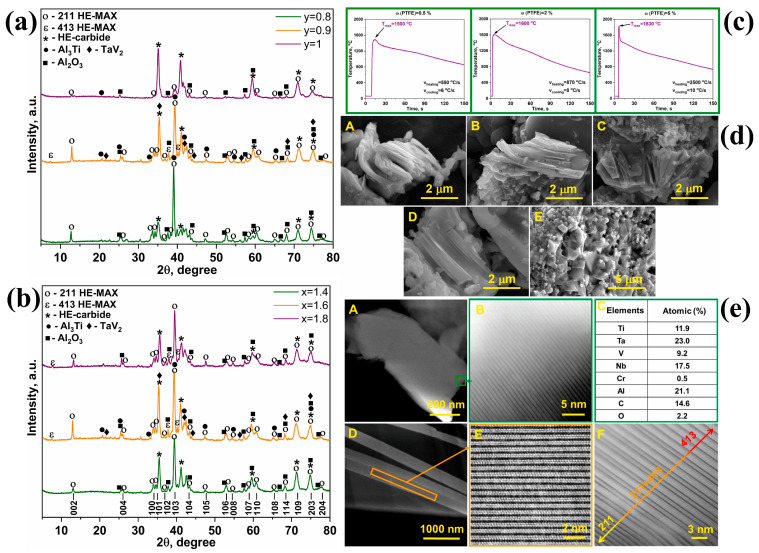
SHS synthesis pathway and phase evolution of (Ti,Ta,V,Nb,Cr)_2_AlC high-entropy MAX phase: (**a**,**b**) XRD patterns showing the influence of Al excess and carbon deficiency on phase formation; (**c**) time-temperature profiles of samples containing PTFE (0.5%, 2%, 5%); (**d**) SEM photographs of the combustion products from 0.4Ti + 0.4Ta + 0.4V + 0.4Nb + 0.4Cr + xAl + yC mixtures, when x = 1.4, y = 0.9 (**A**); x = 1.6, y = 0.9 (**B**); x = 1.8, y = 0.9 (**C**); x = 1.6, y = 0.8 (**D**); x = 1.6, y = 1 (**E**); (**e**) STEM and EDX analysis of the combustion product prepared with 0.5 wt% PTFE: (**A**,**D**) show STEM images of the layered/lamellar HE-MAX structure, (**B**,**E**) show well-defined atomic planes characteristic of the 211 MAX structure, (**C**) presents the EDX elemental composition confirming compositional fluctuations and disordered solid-solution formation in the M layer, and (**F**) shows distinct 211 and 413 stacking sequences as well as alternating 211/413 stacking. Adapted from Figures 1–3, 5, and 8 of [16] under CC BY.

Green (compact) density is another important kinetic parameter. If the compact is too porous, limited particle–particle contact can interrupt reaction percolation and destabilize the combustion front; conversely, overly dense compacts facilitate excessive heat loss from the reaction zone, making ignition and steady propagation difficult. In HE-MAX SHS, this “compact/heat-loss” effect is often practically tuned via the thermal environment and geometry, which directly shapes cooling rate and therefore stacking/microstructure. For instance, during SHS of (Ti,Ta,V,Nb,Cr)_2_AlC, increasing the inert-gas pressure from 0.5 to 3 MPa Ar keeps the combustion temperature around ~1500 °C but changes the combustion wave velocity (0.74–1.33 mm⋅s^−1^) and reduces the cooling rate by ~3×, shifting the morphology from flake-like chains to a stacked laminate structure and increasing the relative contribution of 413-type stacking within a 211/413 mixture (Figure 6). Kinetic control can also be achieved by introducing HE-MAX-relevant active additives that modify mass transport in the reaction zone. In the same system, adding PTFE (0.5–5 wt. %) raised the combustion temperature from ~1500 to 1830 °C and increased the wave velocity from 1.45 → 1.65 → 25 mm⋅s^−1^ (0.5%, 2%, 5%), with 5 wt. % causing thermal explosion; importantly, the product chemistry followed a clear kinetic threshold: >1600 °C favored HE carbide solid solutions, while 1500–1600 °C was more conducive to HE-MAX formation, and moderate PTFE levels could suppress unwanted stacking contributions (e.g., disappearance of 413 at 2% PTFE) [16]. In conclusion, for HE-MAX SHS the most practical kinetic levers after stoichiometry are those that control the thermal history (cooling rate/dwell near the MAX-forming window) and enhance/redirect mass transport (active additives or transient liquid phases), thereby suppressing parasitic HE carbide formation and steering toward layered HE-MAX microstructures.

### 2.4. Compositional Design

The selection of constituent elements is the most critical step in the design of high-entropy MAX (HE-MAX) phases. For the M site (transition metal sublattice), the central objective is to combine four or more metals that can be statistically accommodated within a single MAX lattice, rather than segregating into competing ordered MAX phases or secondary compounds. As a first-pass screening strategy, empirical design principles originally developed for high-entropy alloys (HEAs) can be adapted to HE-MAX systems.

These heuristics emphasize the importance of minimizing atomic size mismatch-quantified by the parameter δ, maximizing the stabilizing contribution of configurational entropy relative to the enthalpy of mixing, commonly expressed by the parameter Ω. Widely cited HEA guidelines suggest that disordered solid solution formation is favored when δ ≤ 6.6% and Ω ≥ 1.1. Because these criteria were formulated for metallic solid solutions, they should be regarded as necessary but not sufficient conditions for HE-MAX formation, serving primarily as a compositional screening tool that must be validated through MAX-specific thermodynamic analysis, phase stability calculations, and experimental synthesis [46,47].

In practical terms, this approach favors M-site combinations composed of transition metals with comparable atomic radii, similar electronegativities, and compatible bonding characteristics. Large disparities in atomic size or bonding tendency promote chemical ordering, phase separation, or decomposition into binary or ternary compounds, undermining the formation of a single-phase HE-MAX structure [48]. Consistent with this view, theoretical stability analyses indicate that disordered HE-MAX phases are preferentially stabilized when the A-group element is relatively large and the mixed M elements exhibit limited size disparity; conversely, small A elements or large M-site size mismatches favor ordered MAX variants or multiphase assemblages [49,50].

The A site is an issue in combustion/SHS synthesis as well since the constituents of many of these A elements (e.g., Al, Si, Zn) can evaporate during rapid high-temperature reaction, giving excess stoichiometry and secondary phases. A usual solution is to manage its A-element supply based on its volatility and reaction pathway. High excess Al content is an important factor to adjust in Al-based MAX phases, 20 mol% of excess Al increased the yield in Ti-Al-C SHS from 56.3 wt% Ti_3_AlC_2_ up to 80.3 wt%; further addition of TiC above this excess Al amount led to even higher yields of synthesized product (89.3 wt% maximal reported) through promoting a stepwise pathway of formation via TiC-related intermediates. For Si-containing MAX, (1) excess A can also provide another means to compensate for evaporation loss, and transient liquid formation promotes rapid diffusion: in Ti_3_SiC_2_ combustion synthesis, 20 mol% excess Si was found to improve Ti_3_SiC_2_ formation by compensating for lost Si at high T, with small amounts of Al addition further increasing the Ti_3_SiC_2_ yield by facilitating liquid phase-assisted transport; authors note that their measured front temperatures exceed the observed Ti-Si eutectic temperature (~1330 °C), which is consistent with a solid–liquid reaction step. When A elements are very volatile (e.g., Zn), direct combustion paths can be replaced by low-temperature exchange chemistry: Ti_3_AlC_2_ can react with molten ZnCl_2_ at 550 °C to form Ti_3_ZnC_2_ (with Zn replacing Al), representing a practical path far below the temperatures at which open SHS processing becomes problematic with respect to retaining significant quantities of Zn [12,19,51]. Another straightforward strategy is operating under elevated inert-gas pressure (e.g., Ar) in a sealed reactor, which can reduce volatilization and support MAX formation. In Cr_2_AlC-based SHS, combustion under ~5 MPa Ar has been reported to improve aluminum retention and increase the MAX-phase fraction, and related studies commonly operate at 5 MPa Ar and report products dominated by Cr_2_AlC under optimized conditions [52,53].

Complementary to pressure containment, compositional design can actively exploit low-melting A-site elements (such as Sn, In, or Ga) to enable liquid-metal-assisted SHS (LMA-SHS). In [15], a novel liquid-metal-assisted self-propagating high-temperature synthesis (LMA-SHS) route for the efficient production of sulfur-containing high-entropy MAX (HE-MAX) phases, addressing long-standing challenges associated with S volatilization and sluggish kinetics in traditional synthesis methods, was reported. By incorporating a low-melting metal such as Sn or In into the SHS precursor mix, the additive melts early in the reaction and acts as a transient liquid binder among transition metal atoms on the M-site, lowering effective mixing enthalpies and significantly enhancing both mass and heat transport during combustion wave propagation.

**Figure 7:**
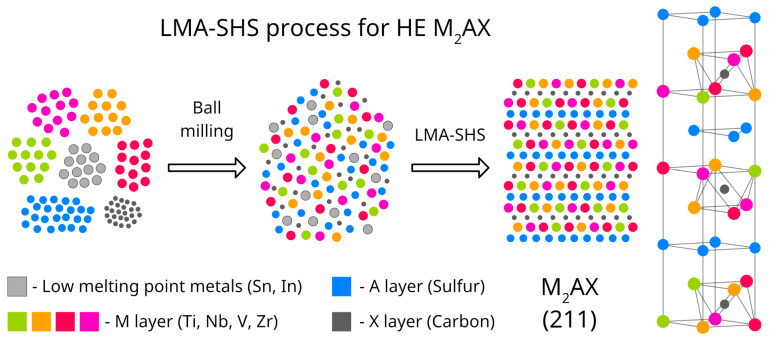
Schematic illustration of the liquid-metal-assisted SHS (LMA-SHS) process for synthesizing (TiNbVZr)_2_(SSn)C. Created by the authors based on the concept reported in [15].

For example, this LMA-SHS strategy was applied to synthesize a sulfur-containing high-entropy MAX phase while mitigating sulfur loss caused by its low boiling point (~444 °C) (Figure 7). In this approach, a fusible metal (Sn or Sn-In; melting temperature ~232 °C) is introduced into the Ti-Nb-V-Zr-S-C reactant mixture, forming a transient molten phase that improves wetting and diffusivity and helps retain sulfur within the reaction zone during rapid combustion. As a result, the high-entropy MAX phase (TiNbVZr)_2_(SSn)C (a high-entropy analogue of Ti_2_SC, with sulfur (and partially Sn) occupying the A-site) can be produced within seconds after filament ignition under an inert atmosphere [15].

## 3. Advantages and Challenges of SHS for HE-MAX Synthesis

Self-propagating high-temperature synthesis (SHS) offers several compelling advantages for the synthesis of high-entropy MAX (HE-MAX) phases. The intrinsically rapid heating rates and short reaction durations promote configurational disorder on the M-site while limiting long-range diffusion, thereby suppressing chemical ordering and the formation of competing binary or ternary phases.

SHS is also highly energy-efficient, as the reaction is sustained by its own exothermicity after ignition, enabling rapid, scalable production with minimal external energy input. In addition, the localized high temperatures achieved at the combustion front facilitate complete reaction and phase formation even in compositionally complex HE-MAX systems, while the flexibility of precursor selection allows for the incorporation of multiple transition metals and non-stoichiometric additives to tailor reaction intensity and phase stability.

Beyond processing efficiency, SHS tolerates complex and non-equilibrium chemistry. The non-equilibrium thermal cycle intrinsic to SHS can be thermodynamically favorable for the formation of high-entropy solid solutions. At elevated temperatures, the −TΔS contribution to the Gibbs free energy lowers the free energy of mixing, increasing the driving force for multicomponent M-site disorder relative to competing ordered phases. Thermodynamic assessments of quaternary and higher-order HE-MAX systems confirm that the combined enthalpy and configurational entropy contributions stabilize the HE-MAX phase field. In addition, SHS is often described as exhibiting a “self-purification” tendency, while the rapid post-combustion cooling suppresses grain coarsening, leading to fine-grained and chemically homogeneous microstructures [12,16,47].

At a practical, engineering-relevant level, the attractiveness of self-propagating high-temperature synthesis for MAX phases comes down to a combination of speed, efficiency, scalability, and compositional flexibility that is difficult to match with conventional routes. Increasing reaction volume raises the volume-to-surface ratio, reducing relative heat losses and promoting higher combustion temperatures, faster front velocities, improved phase conversion, and enhanced homogeneity. This makes SHS attractive not only for laboratory discovery but also for scale-up and industrial production, where furnace time and energy cost dominate [54].

Moreover, SHS offers exceptional compositional tunability. Reaction intensity can be adjusted through stoichiometry, diluents, transient liquid additives, or compact design, allowing practitioners to stabilize reactions near the propagation boundary or moderate overly vigorous combustion. This flexibility enables synthesis of MAX phases that are difficult to access by hot pressing or spark plasma sintering, including compositions containing volatile or sluggishly reacting elements.

Despite these advantages, SHS presents distinct challenges for HE-MAX synthesis that arise from the tight coupling between thermodynamics, kinetics, and heat transfer. The feasibility window for stable combustion is often narrow, particularly for compositions near the propagation threshold where the adiabatic combustion temperature only marginally exceeds the minimum required to sustain the reaction front.

Furthermore, the multicomponent nature of HE-MAX systems increases the likelihood of transient phase separation, uneven element distribution, or volatility-driven losses of A-site species, especially for low-melting or low-boiling constituents. For instance, aluminum melts early during combustion and exhibits high vapor pressure at SHS temperatures, such that evaporation-driven Al loss can shift the effective stoichiometry and substantially reduce MAX-phase yield. Accordingly, a modest excess of the A element (typically 5–20%) is commonly employed to compensate for volatilization. In practice, SHS reactions are conducted under inert or pressurized inert atmospheres, and for HE-MAX systems in particular, the combination of ambient gas pressure, starting composition, and additive selection has been shown to be decisive in determining whether the targeted HE-MAX phase forms [12,16]. Beyond inert-gas and sealed-reactor processing, recent studies suggest that molten-salt shielding can enable selected MAX-phase syntheses under ambient air conditions. For example, the SHS process in the V_2_O_5_,Mg,Al,C system carried out in molten NaCl enabled the formation of V_2_AlC (≈95%), where the molten salt medium played a crucial protective role [55]. Similarly, in [56], synthesized V_2_AlC from V_2_O_5_, Al, and graphite in natural air using a eutectic NaCl/KCl mixture, which melted during combustion and acted as a protective shell that restricted oxygen access to the reacting compact. Although demonstrated for V_2_AlC rather than HE-MAX, this result highlights a promising low-cost route for reducing atmosphere-control requirements during scale-up.

Achieving high phase purity and chemical homogeneity presents an additional challenge because the reaction front evolves rapidly and is strongly sensitive to initial powder/compact conditions. In Ti-Al-C systems, often treated as model systems for understanding MAX-phase SHS behavior, products frequently consist of mixtures of Ti_3_AlC_2_,Ti_2_AlC and TiC, with relative phase fractions strongly dependent on stoichiometry, particle size, green density, mechanical activation, and related variables. These sensitivities underscore the strong thermodynamic competitiveness of TiC and the narrow kinetic window required for selective MAX-phase formation. Such issues are further amplified in HE-MAX compositions due to increased compositional complexity. Phase purity is particularly critical when SHS-derived MAX powders are used as precursors for MXene synthesis: while HF-based etching readily removes carbides and intermetallic, secondary MAX phases (e.g., Ti_2_AlC vs. Ti_3_AlC_2_) persist and can introduce variability in MXene structure, surface chemistry, and functional performance [13,57,58].

Porosity is another inherent limitation of gasless SHS. Green compacts are necessarily below theoretical density, and the combustion event is too brief to permit substantial densification during reaction. Consequently, as-synthesized MAX products are often highly porous and unsuitable for structural applications without further processing. Pressure-assisted SHS and thermal-explosion-based reactive forging provide effective solutions, enabling densification while transient liquid or highly deformable phases are present. For example, reactive forging during thermal explosion has been shown to convert otherwise porous Ti_2_AlC into near-fully dense material under moderate applied loads [13,26].

From a thermal perspective, sustaining SHS requires that the rate of heat generation exceeds heat losses to the surroundings. As a result, measured combustion-front temperatures are invariably lower than calculated adiabatic values. At the same time, the high temperatures required to sustain self-propagation may exceed the equilibrium stability limit of the MAX phase, necessitating rapid cooling to suppress decomposition. Excessive heat sinks, such as overly high green density, aggressive dilution, or excessive tooling contact, can prevent ignition or arrest propagation altogether. Conversely, controlled preheating or thermochemical coupling to auxiliary exothermic reactions can extend the processing window and enable SHS in borderline systems [30,59,60]. Thermally coupled SHS reactions offer a promising route to access HE-MAX phases that are otherwise difficult to synthesize due to insufficient intrinsic exothermicity. In the case of PTFE-assisted activation, both metallic and oxide precursors can be employed. For metallic systems, PTFE promotes the process through highly exothermic metal–fluorine interactions and the formation of gaseous intermediates, enhancing mass transport and facilitating reaction propagation. Additionally, it contributes to oxygen removal by reducing surface oxide layers on metals, leading to cleaner reaction products. In contrast, aluminothermic reduction of oxide precursors represents a more cost-effective approach due to the availability of raw materials; however, it is often accompanied by the formation of secondary phases such as Al_2_O_3_ and metal aluminates. These by-products can be difficult to eliminate, particularly at high temperatures where partial melting of both target and secondary phases may occur. Nevertheless, this approach may remain advantageous for the synthesis of composite materials.

Stable SHS processing of MAX and HE-MAX phases requires coordinated control of composition (including diluents and A-site excess), atmosphere, and thermal management to maintain a self-sustaining combustion temperature while minimizing volatile-element loss. Ambient gas pressure influences both combustion temperature and reaction kinetics, but its role in suppressing evaporation is indirect, operating primarily through its influence on heat losses and reaction-front stability rather than direct vapor-pressure suppression and is therefore highly system-dependent. In contrast, pressure-assisted thermal explosion and reactive forging primarily leverage mechanical pressure to promote densification during or immediately after reaction, rather than as a direct means of controlling A-site volatility [13,61]. Notably, for Ti_3_AlC_2_, thermal-explosion modes can yield higher MAX-phase fractions than steady front propagation, while pressure-assisted variants are widely used to improve density and microstructural uniformity [12].

Under scale-up, the effective SHS stability window is further shaped by heat losses associated with tooling, sample geometry, and containment. Processing studies highlight that while SHS is fast and scalable, steep thermal gradients and high combustion velocities can exacerbate porosity and secondary phase formation unless densification is applied immediately after reaction. Recent Ti-Si-C studies further demonstrate that combustion behavior is sensitive to the reactive-medium structure, including free volume above the compact and granulation or segmentation of the charge, which alter gas transport, burning velocity, and front stability [35,62]. During scale-up, heat and mass transport become increasingly important. Argon backfilling or partial sealing, uniform powder packing, and controlled ignition strategies help minimize lateral heat loss and A-site element volatilization. For systems containing highly volatile components, liquid-metal-assisted SHS (LMA-SHS), previously noted for its kinetic advantages, provides a particularly effective mitigation strategy by introducing a transient liquid phase that enhances wetting, short-range diffusion, and compositional homogenization during the ultrafast reaction. This approach has enabled the synthesis of high-purity (TiNbVZr)_2_(SSn)C within seconds, despite the extreme volatility of sulfur [9].

Two containment/compaction-based strategies are especially promising for improving SHS robustness and scalability in MAX/HE-MAX. First, SHS rapid post-combustion pressing, often at stresses on the order of hundreds of MPa can substantially reduce open porosity and may indirectly limit A-site loss by shortening the duration of exposure to extreme temperatures. Seminal work on pressure-assisted thermal explosion/“reactive forging” work further demonstrated that that applying load during or immediately after combustion yields high Ti_3_AlC_2_ fractions with fine laminated microstructures [35,63].

## 4. Perspectives and Applications

### 4.1. Scalability for Bulk and Coatings

SHS offers rapid conversion (seconds–minutes) and low external energy input after ignition, making it attractive for producing MAX/HE-MAX powders and bulk monoliths. Scaling to larger charges depends on maintaining a stable combustion front as cross-section increases, while also mitigating A-element loss (e.g., Al) through containment strategies such as inert-gas backpressure, partial sealing, and careful control of packing and heat losses. Pressure-assisted combustion and immediate post-front consolidation have been reported to increase density and MAX-phase fraction in Ti_3_AlC_2_, providing a practical framework that can be adapted to larger-scale HE-MAX processing when coupled to uniform packing and controlled quenching [1,13]. Liquid-metal-assisted SHS (LMA-SHS) further widens the process window by introducing a transient Sn/In melt that improves wetting and short-range homogenization during the thermal spike, enabling high-purity HE-MAX formation with reduced effective residence at peak temperature. This is particularly attractive for volatile or compositionally complex systems and for scale-out to larger batch geometries [15]. For coatings, SHS-derived powders can serve as feedstock for thermal spray or cold spray routes, while substrate-integrated combustion (thermal-explosion mode) can form adherent MAX layers in situ. Recent overviews summarize processing routes and the resulting oxidation/wear behaviors of MAX-based coatings and overlays [14].

### 4.2. Integration into Derivative Phases (MXenes)

HE-MAX phases synthesized by SHS are attractive precursors for high-entropy Mxenes primarily because the self-sustaining nature of the process enables the rapid, energy-efficient production of complex multicomponent ceramics that would otherwise require prolonged high-temperature furnace treatments. While rapid high-temperature exposure followed by fast cooling characteristic of SHS can help to preserve multi-principal M-site occupancy before selective A-layer removal. Recent surveys emphasize the expanding pathway from composition-engineered MAX phases to 2D carbides/nitrides with tunable conductivity, surface chemistry, and catalytic/energy-storage behavior. In electrocatalysis (HER/OER), multi-metal synergy together with defect/terminal group control is frequently highlighted as a route to improved activity and stability, and HE-MXenes derived from scalable MAX synthesis routes could benefit from these design levers [4,64].

### 4.3. Potential Functional Properties

**Figure 8:**
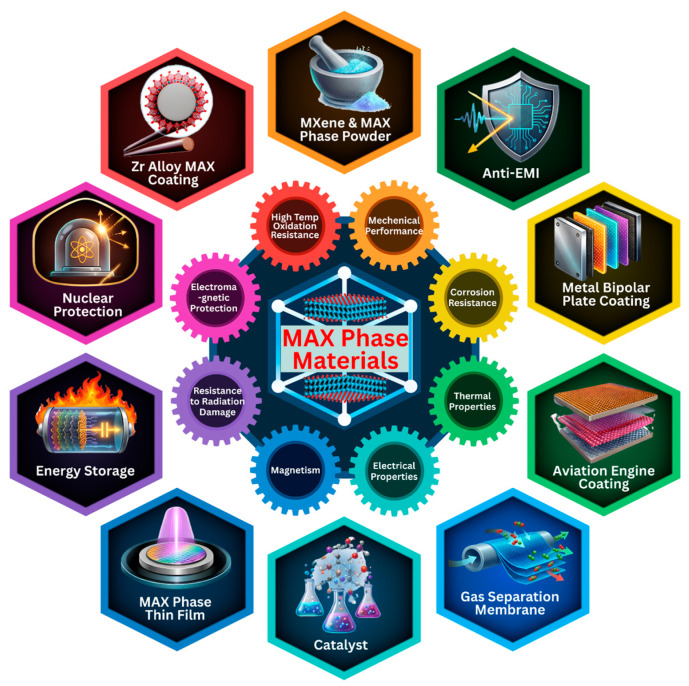
Properties and applications of MAX phase materials.

MAX phases already occupy an unusual position between metals and ceramics, combining high stiffness with damage tolerance and machinability. For SHS-processed materials, ultrafast thermal cycles can yield refined lamellar microstructures and limited grain growth, while pressure-assisted variants improve density and interlamellar integrity, which is beneficial for wear-resistant coatings and structural components. For high-temperature environments, layered MAX coatings can offer thermal-shock resistance and oxidation protection; HE chemistries further open opportunities to tailor oxide scale formation and diffusion barriers. In electronic/transport domains, compositional complexity at the M-site provides levers to tune conductivity and scattering, enabling applications such as EMI shielding, contact layers, and high-temperature electrodes. Overall, assisted SHS modes, including LMA-SHS, provide a rapid and energy-lean route to bulk HE-MAX powders and dense parts, while also supporting scalable precursor supply chains for HE-Mxenes [1]. Various properties and applications of MAX phase materials have been illustrated in Figure 8.

## 5. Conclusions and Outlook

The emergence of high-entropy MAX (HE-MAX) phases holds profound fundamental research significance by extending MAX phase chemistry from conventional ternary systems to a vast, multidimensional compositional space governed by entropy-driven stabilization. These materials provide a unique platform to investigate the interplay between long-range crystallographic order and short-range chemical disorder within anisotropic, layered ceramics—an area that remains largely unexplored. HE-MAX phases enable fundamental insights into configurational entropy effects, the competition between enthalpy and entropy in phase formation, and the role of chemical complexity in determining electronic structure, defect behavior, and transport properties. Furthermore, they bridge the conceptual gap between high-entropy materials and nano-laminated ceramics, offering a model system for studying disorder-induced phenomena in structurally ordered frameworks. Beyond their intrinsic scientific value, HE-MAX phases also serve as precursors to high-entropy MXenes and multifunctional materials for emerging applications in energy, catalysis, and extreme environments.

The convergence of high-entropy (HE) materials chemistry with self-propagating high-temperature synthesis (SHS) represents a transformative opportunity to redefine the accessible compositional and structural landscape of MAX phases. As highlighted throughout this review, SHS provides a uniquely powerful platform for HE-MAX synthesis by coupling extreme yet highly localized thermal fields with ultrafast reaction kinetics, enabling access to multicomponent, metastable, non-equilibrium phases that remain largely inaccessible through conventional long-dwell processing routes.

The feasibility of SHS-derived HE-MAX phases is governed by a tightly interwoven thermodynamic–kinetic window, wherein reaction enthalpy sustains combustion wave propagation in the presence of inevitable heat losses, configurational entropy stabilizes high-temperature multicomponent solid solutions, and rapid heating and quenching suppress equilibrium phase separation while promoting homogeneous chemical disorder. Within this framework, precursor powder characteristics, green compact design, stoichiometry, and the deliberate use of kinetic modifiers such as diluents, transient liquids, or assisted ignition strategies emerge as powerful levers for tailoring reaction stability, phase purity, and microstructural evolution.

Although A-site volatility remains the dominant engineering challenge for many HE-MAX systems, recent advances demonstrate that it is increasingly manageable through rational process design, including modest A-site excess, inert-gas containment or backpressure, pressure-assisted compaction, and liquid-metal-assisted SHS routes. These developments underscore the growing maturity of SHS as a controllable synthesis methodology rather than a purely empirical combustion process.

Looking forward, the integration of CALPHAD- and first-principles-guided compositional screening with SHS-specific processing maps offers a clear pathway toward predictive design of HE-MAX chemistries. Equally critical will be more rigorous and standardized reporting of key SHS metrics—such as combustion temperature, wave velocity, and heat-loss conditions—as well as quantitatively grounded scale-up studies that bridge laboratory demonstrations with industrial relevance. Together, these advances position SHS not only as a discovery tool, but as a scalable, energy-efficient manufacturing route for HE-MAX precursors, accelerating the realization of high-entropy MXenes and their deployment in next-generation structural, thermal, and electrochemical applications.

## Figures and Tables

**Table 1:** Processing–structure relationships in SHS/TE synthesis of MAX phases.

Processing Variable	Low/Insufficient	Optimal Window	Excessive/Destabilizing
Effective exothermicity (Tad)	No ignition or quenched front due to heat losses	Self-sustaining SHS or controlled TE enabling MAX/HE-MAX formation	Overheating, decomposition, A-site evaporation
Diluent fraction/intermediate reactants	Poor ignition, incomplete reaction	Moderated combustion temperature and stable propagation	Excess heat sink, front extinction
A-site content (e.g., Al)	Off-stoichiometry, TiC-rich products	5–20% A-site excess compensates volatility	Excess liquid, segregation, secondary phases
Green density/particle size	Poor particle contact/percolation, unstable front	Stable propagation with controlled heat extraction	Difficult ignition, arrested propagation
Atmosphere (vacuum/inert gas pressure)	Enhanced A-site loss, surface decomposition	Controlled volatility and reaction kinetics	Suppressed propagation or altered kinetics (system-dependent)
Combustion mode	No propagation/partial reaction	Propagating SHS or thermal explosion (TE)	Uncontrolled runaway
Mechanical pressure (if applied)	Porous products	Reactive forging/post-front densification	Premature quenching if applied too early
Cooling rate	Slow cooling → decomposition	Rapid quench preserves MAX/HE-MAX	Incomplete conversion/retained intermediates (system-dependent)
Outcome	Mixed phases, high porosity	High-purity, fine-grained MAX/HE-MAX	Decomposition, loss of target phase

Processing map for SHS of MAX phases. Compiled from [12,13,19,26,28,29,30,33,34,35].

**Table 2:** SHS control parameters.

Control Parameter	Practical Guideline	Consequence if Violated
Adiabatic temperature (Tad)	Ensure Tad-heat losses ≥ propagation threshold	No ignition or quenched front
Preheating/trigger reactions	Use for borderline systems	Poor ignition reliability
Powder size and dispersion	Fine, well-mixed powders	Diffusion-limited reactions, mixed phases
Green density	Intermediate (contact without excessive heat sink)	Excess heat loss or poor propagation
Diluents/modifiers	Introduce conservatively; optimize via Tc and velocity	Quenched propagation, low yield
A-site stoichiometry	5–20% excess to offset volatility	A-site loss, carbide dominance
C/metal ratio	Near stoichiometric (slight deficiency only if justified)	Reduced heat release, low conversion
Atmosphere/sealing	Inert gas, partial sealing for scale-up	A-site evaporation, instability
Pressure assistance	Apply during/after combustion for densification	Premature quenching if applied early
Liquid-metal assistance	Use for volatile/complex HE-MAX systems	Poor homogenization, impurity phases

## Data Availability

No new data were created or analyzed in this study. Data sharing is not applicable to this article.
